# Examining the readiness of best evidence in medical education guides for integration into educational practice: A meta-synthesis

**DOI:** 10.1007/s40037-018-0450-9

**Published:** 2018-09-18

**Authors:** Lauren A. Maggio, Aliki Thomas, H. Carrie Chen, John P. A. Ioannidis, Steven L. Kanter, Candace Norton, Nancy H. Tannery, Anthony R. Artino Jr.

**Affiliations:** 1 Uniformed Services University of the Health Sciences, Bethesda, MD USA; 2 McGill University, Montreal, ON Canada; 3 Georgetown University School of Medicine, Washington, DC USA; 4 School of Medicine and Meta-Research Innovation Center at Stanford (METRICS), Stanford University, Stanford, CA USA; 5 School of Medicine, University of Missouri-Kansas City, Kansas City, MO USA; 6 National Institutes of Health, Bethesda, MD USA; 7 University of Pittsburgh, Pittsburgh, PA USA

**Keywords:** Knowledge syntheses, Literature review, Knowledge translation

## Abstract

**Background:**

To support evidence-informed education, health professions education (HPE) stakeholders encourage the creation and use of knowledge syntheses or reviews. However, it is unclear if these knowledge syntheses are ready for translation into educational practice. Without understanding the readiness, defined by three criteria—quality, accessibility and relevance—we risk translating weak evidence into practice and/or providing information that is not useful to educators.

**Methods:**

A librarian searched Web of Science for knowledge syntheses, specifically Best Evidence in Medical Education (BEME) Guides. This meta-synthesis focuses on BEME Guides because of their explicit goal to inform educational practice and policy. Two authors extracted data from all Guides, guided by the 25-item STructured apprOach to the Reporting In healthcare education of Evidence Synthesis (STORIES).

**Results:**

Forty-two Guides published in *Medical Teacher* between 1999 and 2017 were analyzed. No Guide met all STORIES criteria, but all included structured summaries and most described their literature search (*n* = 39) and study inclusion/exclusion (*n* = 40) procedures. Eleven Guides reported the presence of theory and/or educational principles, and eight consulted with external subject matter experts. Accessibility to each Guide’s full-text and supplemental materials was variable.

**Discussion:**

For a subset of HPE knowledge syntheses, BEME Guides, this meta-synthesis identifies factors that support readiness and indicates potential areas of improvement, such as consistent access to Guides and inclusion of external subject matter experts on the review team. This analysis is useful for understanding the current readiness of HPE knowledge syntheses and informing future reviews to evolve so they can catalyze translation of evidence into educational practice.

## What this paper adds

By analyzing BEME Guides, a collection of HPE knowledge syntheses, this meta-synthesis identifies and characterizes factors, related to the quality, accessibility, and relevance of the Guides, that support readiness for translation into educational practice. Our findings highlight positive practices, such as including structured summaries, well-described literature searches, and rationale for undertaking knowledge syntheses. However, there is room for improvement related to accessibility and relevance, such that those that author and publish Guides, as well as similar publications, might consider increasing stakeholder participation, ensuring consistent and public access to Guides, and incorporating relevant educational theory.

## Introduction

In health professions education (HPE), researchers have argued that knowledge syntheses are as important as primary studies [1]. Thus, it is unsurprising that their numbers have been on the rise, with most HPE journals accepting knowledge syntheses, and foundations and professional groups offering funding for their creation (e.g., the Gold Foundation). Additionally, for almost 20 years, the Best Evidence in Medical Education (BEME) Collaboration has supported HPE researchers in conducting and disseminating knowledge syntheses, which are known as BEME Guides.

Despite support for their creation and swelling numbers [2], knowledge syntheses, especially systematic reviews, have been criticized not only by those who try to use them, but also by those who publish them [1, 3–5]. Norman argued that many reviews end up as exercises in ‘bean counting’ and often fail to provide conclusive, usable evidence for practitioners [6]. Furthermore, Gordon suggested that systematic reviews often lack relevance and fail to incorporate educational elements critical for educational practice and policy [1]. Despite these criticisms, our field has yet to evaluate HPE knowledge syntheses to determine whether or not they are ready for translation into educational practice and policy.

HPE is not unique in its lack of understanding about the readiness of knowledge syntheses [7]. In clinical medicine, for example, Glasziou reported that systematic reviews of non-pharmacological interventions lacked details to translate findings into practice, such that it was impossible to determine which versions of treatments to use [8]. More recently, a study of over 50 systematic reviews of stroke interventions identified that 80% were missing key details, such as intervention procedures and materials [9]. In education, this might be akin to a knowledge synthesis evaluating the use of simulation, but not clarifying the strategies that may be deployed for optimal teaching and assessment within simulation.

Without understanding the readiness of our knowledge syntheses, defined by three criteria—quality, accessibility and relevance—we risk translating weak evidence into practice and/or providing information to educators that is not usable, both of which have the potential to frustrate teachers and learners and impair teaching and learning practices. Thus, this article is a first attempt to identify and characterize factors that support the readiness of HPE knowledge syntheses for translation into educational practice. In this study we chose to focus on BEME Guides because they are ‘designed to assist individual teachers, institutions, and national bodies to make informed decisions about educational practice and policy’[10]. In other words, Guides are explicitly meant to inform educational practice and policy and are described as ‘user-friendly’ such that practitioners can assess and apply them in a manner appropriate for their own criteria and context [11].

## Method

We analyzed HPE knowledge syntheses, specifically BEME Guides (hereafter referred to as ‘Guides’), to estimate their readiness for integration into practice.

While we recognize that the Guides comprise a minority of HPE knowledge syntheses and that not all knowledge syntheses aim to address the knowledge-to-practice gap, we focused on the Guides based on their specific mission to inform practice and policy and to be useful to teachers, researchers and policy makers. Additionally, we focused on Guides because their authors are required to follow structured instructions for the creation of the review, which we believed would allow us to draw comparisons across the Guides.

We convened an author team of experts in HPE, knowledge syntheses, knowledge translation, clinical medicine, information science, scholarly communication, and health policy. Our team also has experience as researchers and as teachers of health professional learners in classrooms and clinics. The research team was convened to maximize expertise in HPE educational practice and research, as well as expertise in knowledge synthesis.

**Fig. 1:**
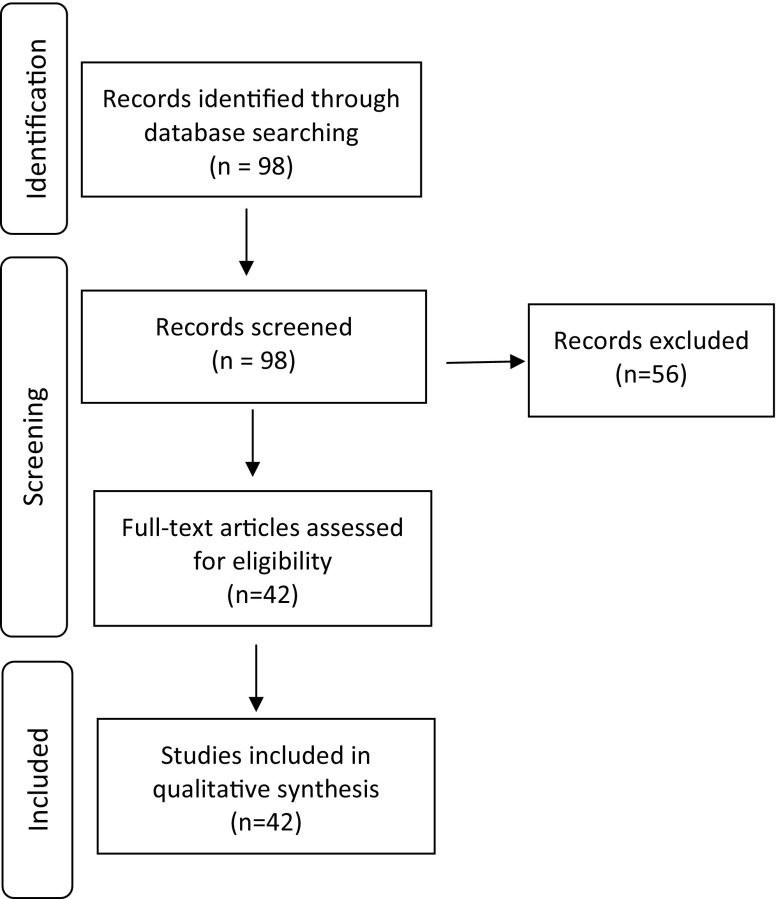
Flow diagram of the identification, screening and identification of studies

To identify Guides, on 1 May 2017, we searched Web of Science using ‘BEME’ as a keyword. We downloaded the identified citations into an Excel spreadsheet. We also checked the BEME website (https://www.bemecollaboration.org/) to confirm that we had retrieved all the Guides. Based on titles and abstracts, we excluded non-review citations (e.g., articles about BEME’s future, protocols, or articles on how to write a Guide; see Fig. 1). From *Medical Teacher*, we obtained the full-text of all included reviews in PDF form. When necessary and as possible, we also obtained the text of online supplements from the *Medical Teacher* and/or the BEME websites.

Data extraction was guided by the STructured apprOach to the Reporting In healthcare education of Evidence Synthesis (STORIES) Statement [12]. The STORIES Statement, which includes 25 elements, is a consensus statement on the baseline reporting requirements for knowledge syntheses. In addition to coding the 25 elements, coders kept descriptive notes with an emphasis on identifying exemplars. We selected the STORIES Statement, instead of a more generic methodological tool, such as AMSTAR[13], because STORIES is designed specifically for HPE by HPE researchers and includes items specific to education (e.g., identifying the use of pedagogical strategies). Additionally, it supports the varied question types HPE researchers pose, which often go beyond determining an intervention’s effectiveness. For example, one Guide characterized why underperforming learners do not fail [14] and another described what physicians need to know about ecosystems [15]. Lastly, we selected the STORIES Statement since it does not focus on one specific type of knowledge synthesis but instead is flexible and applicable to all knowledge synthesis types.

Two reviewers extracted data from all the Guides. LM coded all the Guides, while CN, HCC, and NT each independently coded 14. Discussions between the coders occurred before, mid-way through, and after coding completion. At each point, the authors compared codes for shared understanding. If two coders disagreed, a third acted as a tiebreaker. Upon overall consensus, LM compiled all data extractions into a master spreadsheet, which was emailed to the entire study team.

## Results

We identified 42 Guides published in *Medical Teacher* between 1999 and 2017. Thirty-six Guides were described as systematic reviews [14–49], three as reviews [50–52], two as realist reviews [53, 54], and one as a review with quality grading of articles [55]. Most authors rationalized conducting their Guides due to a lack of knowledge synthesis covering their specific topic. However, there was limited justification as to why a specific synthesis type was selected and whether or not this selection aligned with the Guide’s research question (See Tab. 1 for an overview of the review characteristics; See Tab. 2 for methodological details). To structure the reporting of our findings, we use the concept of readiness criteria as defined by three criteria: quality, accessibility and relevance. These criteria are derived from the first BEME Guide published by Harden [56].

**Table 1:** Review characteristics

Average number of included primary studies	53 (Range 5–415)
Populations included in reviews	Single profession focus: 25 studies Multiple professions focus: 17 studies Medicine: 34 Nursing: 15 Dentistry: 9 Pharmacy: 7 Veterinary: 4 Other: 7 Unspecified: 3
Country of first author	United Kingdom: 17 Canada: 8 Australia:5 United States: 4 Netherlands: 3 Ireland: 1 Bahrain: 1 Denmark: Iran: 1
Number of funded reviews	17

**Table 2:** Inclusion of methodological details

*Identification of studies*	
States and provides a rationale for how the searching was done	Yes—40 (95%) No—2 (5%)
Provides details on all the sources of information and dates searched	Yes—36 (86%) No—6 (14%)
Provides full search terms for at least one database with details of deviations in subsequent searches	Yes, available in full-text^a^—21 (50%) Yes, available in supplement only^b^—13 (31%) No—8 (19%)
*Data extraction*	
Describes the process of data extraction	Yes—39 (93%) No—3 (7%)
Describes the process of contacting authors for confirmation of/or more data	Yes—8 (19%) No—34 (81%)
*Inclusion/exclusion*	
Explains the method for judging inclusion/exclusion	Yes—40 (95%) No—2 (5%)
Describes quality appraisal tools used in data extraction and justifies its use	Yes—41 (98%) No—1 (2%)
Provides a flow diagram summarizing study selection	Yes, available in full-text—28 (67%) Yes, available in supplement only—8 (19%) No—6 (19%)
*Synthesis*	
Describes methods for synthesizing primary studies	Yes, across all studies: 28—(67%) No, across all studies: 14—(33%) Describes qualitative methods: 22 Describes quantitative methods: 6 Describes quantitative and qualitative methods: 4

^a^Materials were considered available in full-text if they were present in the available PDF version from *Medical Teacher*

^b^Materials were considered available in supplement only if they were not featured in the PDF version of the article, but were available on either the BEME or *Medical Teacher* website

### Quality

We considered quality in relation to how each Guide fulfilled the 25 elements of the STORIES Statement. All reviews fulfilled the following four criteria: contained a structured summary, provided rationale for conducting the review, summarized details of the included studies, and presented findings in light of stated objectives. The least satisfied criteria (*n* = 8 Guides) pertained to research teams contacting external individuals familiar with the topic. Below we highlight several key criteria. Detailed information for each criteria is available in Supplement 1: https://figshare.com/s/590bcc368c7609280aec.

#### Research questions

All Guide authors but one [55] stated research questions, generally featured in a Guide’s introduction section and/or set off with bullet points. Fifteen guides reported the Population, Intervention, Comparison, Outcome (PICO) elements of their questions. Most Guides focused on intervention effectiveness (e.g., ‘Is the journal club effective in supporting evidence-based decision making?’ [31]). However, several Guides addressed how to characterize concepts by asking questions, such as ‘What factors have been studied that may be associated to medical-career decision making?’ [40].

#### Literature searching

Thirty-nine Guide authors provided a rationale for how they conducted their literature searches. To a slightly lesser degree, authors detailed their search processes (*n* = 36) and provided the full search terms for at least a single database (*n* = 34). Information professionals were involved in conducting the searches for 16 Guides.

#### Inclusion/Exclusion

All Guide authors, except one [55], described methods for judging the inclusion and exclusion of identified studies and performed data extraction in duplicate. In 36 guides, authors included a flow diagram, such as recommended by the Preferred Reporting Items of Systematic Reviews and Meta-Analyses (PRISMA) Guidelines [57].

#### Qualitative and quantitative analysis

Twenty-three Guides utilized and rationalized their use of qualitative approaches to synthesizing primary evidence, including thematic analysis, qualitative description, and narrative description. Twenty-nine Guides described quantitative methods used to synthesize primary evidence and discussed how they considered the heterogeneity of included studies. Four Guides included meta-analyses [21, 29, 33, 36]. Heterogeneity of the data was the main reason provided for not performing meta-analyses. For example, Reeves pointed out, ‘Due to the heterogeneity of interventions (differing curriculum content, duration of courses, participating professional groups) and study designs (quasi-experimental, exploratory, action-oriented) a meta-analysis of studies was not possible [42].’

### Accessibility

We considered accessibility in terms of readers’ ability to access, either in hard-copy or online, the full-text of a Guide and its supporting materials. Detailed online accessibility information for each article is available in Supplement 1 (Available at: https://figshare.com/s/590bcc368c7609280aec.) Additionally, we examined the steps authors took (e.g., inclusion of practice points) to make Guides cognitively accessible for a variety of readers, including busy clinician-educators who may not be engaged in HPE research as a primary duty.

The full-texts of all Guides are available by subscription in *Medical Teacher*. Of the included Guides, 26 were freely accessible from the BEME website. These free versions tended to be the author’s final draft, meaning that readers may encounter documents with author ‘track changes’ and tables and figures missing or located at the end of the manuscript. On the *Medical Teacher *website, 11 reviews were freely available as of 24 March 2018, and several of them overlapped with those featured on the BEME website. As of 24 March 2018, 11 Guides were not publicly accessible, all of which were published between 2015–2017, thereby comprising the majority of recently published Guides. For most Guides, components of the methods and results, such as search strategies, data abstraction tools, and results tables, were made available on the BEME and/or *Medical Teacher* websites with neither location containing all the materials.

Guides included or made available supplemental materials to increase the cognitive accessibility for readers at differing levels of engagement with HPE research. For example, all Guides featured publicly accessible abstracts. Currently, *Medical Teacher* requires structured abstracts under 200 words. Reviews published from 2008–2017 followed this convention. However, reviews published prior to 2008 were significantly longer and included robust method sections and, in many cases, ‘headline results’ or ‘highlight points’ set off with bullet points. These brief points connected readers with key findings for practice, making the material more readily accessible. For example, one review included the practice point ‘educational feedback is the most important feature of simulation-based medical education [32].’ Additionally, within the body of 35 Guides, authors included practice points. Practice points, generally featured in the Guide’s introduction section, are 4–6 bulleted points, offset in a box, designed to provide readers a brief snapshot of major findings. For example one review noted, ‘It is key that portfolio implementation is well-designed and sustained, with high-level organizational support, to ensure uptake’ [47] and another that ‘The Education for Sustainable Healthcare framework can guide curricula and teaching development [15].’

External to the Guides themselves, 34 authors included a ‘BEME Spotlight.’ A BEME spotlight is a two-page structured summary of the Guide’s main conclusions and recommendations. To host these materials, and others, each Guide has a webpage on the BEME website (https://www.bemecollaboration.org/Published+Reviews/). Page contents vary by Guide, but generally included links to the Guide’s full-text (if publicly available), the review protocol, an executive summary, PowerPoint slides from conference presentations, conference posters, and the *Medical Teacher *website. For example, the BEME site for O’Dunn-Orto’s Guide on the musculoskeletal exam [37] features links to its abstract, a publicly-accessible MS Word version of the full-text, a conference poster, a PowerPoint presentation, and the associated *Medical Teacher *article. While the BEME and *Medical Teacher* websites indicate via links and/or references that these materials exist, if a practitioner were to access a Guide via PDF, there is no indication in the PDF document that these supplemental materials exist.

### Relevance

Relevance is the ‘degree to which something is related or useful to what is happening or being talked about [58]’. Because relevance has a personal dimension, and because a given teaching approach may be relevant to one educator and not to another, we did not judge each Guide’s relevance, per se. Instead, we report on the elements provided that might inform readers in making their own relevance judgments based on their individual practice situations. Elements related to relevance included: implications for researchers and educators, inclusion of stakeholders, inclusion of educational theories and/or models, and currency.

#### Implications for researchers and/or educators

Forty Guides included sections focused on implications for future research that were generally located before the Guide’s general conclusion. Fewer Guides reported implications for education/practice (*n* = 27). For example, the Guide on role modelling included the implication: ‘Role modelling should be explicit in clinical teaching, as it is important for teachers to make an intentional effort to articulate what aspects they are modelling [39].’

#### Inclusion of stakeholders

Authors from a variety of backgrounds participated in the Guides, including, but not limited to, clinicians (*n* = 37 Guides), librarians (*n* = 16 Guides), trainees (*n* = 11 Guides) and statisticians (*n* = 2). Author teams ranged from a single author [55] to 15 authors [38]. The average author team had six members. Nineteen Guides explicitly described how authors’ content and/or methodological expertise benefited the Guide. For example, Issenberg described targeted recruitment of team members with content knowledge across a variety of simulation modalities, skills in educational measurement, and expertise in research methods [32]. External to the author team, 25 Guides engaged subject matter and synthesis experts as consultants. For example, Buckley enlisted a professional translator to translate non-English primary studies and a librarian to design and execute the literature searches [53].

#### Inclusion of theory and educational principles

Eleven Guide authors reported on theories, conceptual frameworks, and/or educational principles that underpinned the individual studies that they had analyzed. For example, Steinert reported how the studies she included adhered to principles of teaching and learning and cited adult learning and experiential learning as organizing concepts [44]. For Guides that did not mention theory or educational principles, it was generally unclear if they were not reported because they were absent from the individual studies or if the review authors chose not to cover these elements. In one Guide, the author notes that theory was absent from the studies analyzed [50].

#### Currency

The oldest Guide, focused on communication skills, was published in 1999 with its most recent study analyzed in 1998 [55]. In contrast, the most recent Guide, published in 2017, analyzed studies up until 2014 [34]. Three Guides had been formally updated [25, 30, 44]. For example, in one instance, 10 years after initial publication, the author revised her earlier Guide on faculty development [43, 44]. The two other Guides were updated 4 [49] and 9 years [42] after the original Guides were published. In the revised Guides, the authors explicitly referenced and built upon their earlier work. In 2013, Cherry posted to the BEME website a brief, publicly accessible ‘follow-up’ report of her review on emotional intelligence 1 year after its publication in *Medical Teacher *[23].

## Discussion

We analyzed 42 BEME Guides to identify and characterize factors that support the readiness of knowledge syntheses for use by HPE practitioners. In our findings, we have highlighted positive practices, such as the universal inclusion of structured summaries. However, despite being considered ‘the gold standard’ of educational reviews [59], no Guide met all the STORIES Statement criteria and there was considerable room for improvement in relation to accessibility and relevance. We now consider readiness criteria in light of the existing literature, highlight best practices, and where appropriate provide suggestions for future HPE knowledge syntheses.

### Quality

Practitioners infrequently apply generic evidence to their educational practice without first considering its fit with their local context [60]. Clearly stated research questions can help practitioners to quickly determine a review’s focus and determine its relevance for their practice. We found that the Guides, with one exception, presented their research questions; however, the minority called out the PICO elements. Common in clinical research and a requirement for reviews following the PRISMA Guidelines [57], PICO elements provide a structured representation of the research question, which may facilitate a reader’s ability to quickly identify its relevance to their context. While we recognize that the ‘kaleidoscopic nature’ of HPE research [61] may give rise to a variety of types of research questions and the use of diverse methodologies, the majority of Guides were systematic reviews, a synthesis type for which PICO questions are quite appropriate. Recently, the BEME Collaboration has promoted the use of a variety of types of knowledge synthesis and provides resources to help researchers explore and adopt the knowledge synthesis methodology most appropriate for their research question [11].

Most Guides reported the details of how they executed literature searching and determined the inclusion and exclusion of studies. By reporting these details, authors provided transparency in their methods thus allowing readers to judge the strengths and weaknesses of the review. Including these details also allows future researchers to fully replicate and update the Guide, thereby avoiding inefficiencies and waste [62].

### Accessibility

In fields such as public health and clinical medicine, practitioners report that the inability to access full-texts of information in a timely and cost-effective manner is a major barrier to uptake of evidence into practice [63]. Fortunately, the full-text of almost 75% of the included Guides was publicly accessible in some form. However, more recent reviews require subscription privileges or payment of $54.00 for 24-hour access. For many educational practitioners, this is a barrier that can potentially impede integrating current evidence into practice. We suggest review authors exercise their right, as described by *Medical Teacher’s* publisher, Taylor & Francis, to deposit a copy of their original manuscript (prior to peer review) to a preprint server [64]. Preprint servers provide practitioners with barrier-free, immediate access to Guide content. Additionally, preprint servers are indexed by search engines, such as Google Scholar, making them findable by a broad audience of educators. Lastly, recent research has reported that articles with an associated preprint actually have higher Altmetric scores and citation rates than those without [65].

The creation of targeted derivative products of the Guides, such as Practice Points and BEME Spotlights, aligns well with HPE practitioners’ requests for straightforward syntheses and brief presentations of empirical evidence [66]. In other disciplines, similar approaches have been linked with increased understanding of evidence and improvements in knowledge uptake and application to practice [67–69]. Therefore, these materials should be made maximally visible and readily accessible. Going forward, *Medical Teacher* editors might consider embedding links to these materials on their website or directly referencing them within the review’s full-text. Additionally, the editors might follow *Academic Medicine’s* example and assemble these valuable resources as an e‑book [70].

### Relevance

In HPE, the transferability of educational approaches and innovations from one institution to another and between learners has been questioned [71]. To address this issue, researchers have suggested that theory-informed approaches enable practitioners to understand why things work and under what conditions, thus making it possible to transform ‘off the shelf’ approaches into approaches appropriate for their own educational contexts [72–74]. Therefore, it is recommended that descriptions of educational interventions report on the principles, theories, and/or philosophies upon which those interventions are based. This idea is quite similar to the advice of Varpio et al., who noted, ‘While an educational innovation’s techniques may seem to be surface structures, they are realizations of deeper fundamental principles. The fundamental principles are themselves realizations of the innovation’s foundational philosophy. When techniques and/or principles are modified to a context, it is important to analyze if the modifications continue to uphold the innovation’s philosophy [74].’ Unfortunately, a minority of Guides reported these elements. We believe this is an area ripe for improvement, which could, in turn, facilitate greater transferability.

BEME guidelines state that a Guide should be updated within 3 years of publication [75]. Currently, few Guides meet this criterion suggesting some findings may no longer be relevant. BEME suggests that authors, in addition to publishing a formal update, post a supplement to the original review that highlights important studies published since the completion of the original review. We identified a single supplementary update [23]. It is the Guide author’s prerogative to update their work (or not); however, BEME reserves the right to permit other researchers to update a Guide. In the future, BEME might consider partnering authors with graduate programs in HPE to enlist students help with increasing the timeliness of Guide updates. Nevertheless, we should also acknowledge that the timing of necessary updates may vary, depending on how much new information is available and whether that new information makes a meaningful difference. Some topics may have no new studies accrue for many years, while others may need very frequent updates [76–78].

### Study strengths and limitations

We focused on a subset of HPE reviews that are highly curated and well-supported. Therefore, these findings may not generalize across HPE reviews. Future research should consider expanding our approach to examine HPE reviews more broadly. We framed our analysis of the Guides using the STORIES Statement. However, future researchers may want to more specifically focus on evaluating the overall quality of the Guides using another targeted tool, such as the AMSTAR Checklist.

Notwithstanding these limitations, we believe this meta-synthesis has a number of important strengths. To our knowledge, it is the first analysis of BEME Guides, which are created for use in educational practice. In addition, we undertook a systematic search and independently engaged in extracting data from all Guides. Lastly, we engaged in this research with a diverse team of stakeholders who have varied expertise and backgrounds. We hope this fact increases this meta-synthesis’ relevance to a variety of stakeholders. Ultimately, we are optimistic that this analysis will help those who write and publish future Guides, and similar publications, continue to evolve such that knowledge syntheses can more fully catalyze the translation of evidence into educational practice.
